# Irrational Beliefs and Personality Traits as Psychological Mechanisms Underlying the Adolescents' Extremist Mind-Set

**DOI:** 10.3389/fpsyg.2019.01184

**Published:** 2019-05-22

**Authors:** Simona Trip, Mihai Ion Marian, Angelica Halmajan, Marius Ioan Drugas, Carmen Hortensia Bora, Gabriel Roseanu

**Affiliations:** Department of Psychology, University of Oradea, Oradea, Romania

**Keywords:** radicalization, extremism, irrational beliefs, personality, psychological mechanisms

## Abstract

The tripartite model of militant extremist mind-set proposed by Stankov et al. (2010b) includes three components: War (justification of violent acts as war); God (violence is seeing extremist acts as moral because they are done in the name of God or Allah); and West (violence against Western countries is justified because they are perceived as evil and/or immoral). There is a lack of conceptual framework regarding psychological mechanism that underlie radicalization and extremism, and there is little evidence regarding risk factors for radicalization in the scientific literature. In the present study, it is hypothesized that irrational beliefs and a constellation of personality factors are two possible psychological mechanisms that put adolescents in a vulnerable position and could influence them to develop an extremist mind-set. The sample consists in 295 Romanian adolescents, ages 15–18 years, and the mean age being 16.41. The present study was conducted in several schools from Bihor County located in the north-western part of Romania. Adolescents took part on a voluntary basis in the study after the written, informed consent was obtained from their parents. A confirmatory factor analysis (CFA) on the structure of Militant Extremist Mind-Set Scale confirmed the three-factor model of the extremist mind-set. Two confirmatory factor analyses were also conducted for the other two administered scales: CASI and Mini-IPIP. The results support the previous models for both scales, including items loading on factors. SEM analysis was performed with AMOS 23 statistical package on a final sample size of 242 participants and there were no missing data. Fifth structural models were specified. The fifth model had adequate fit based on all three indices including the RMSEA (0.054), CFI (0.958), and SRMS (0.047). Global evaluation of self seems to be the only irrational belief that was somewhat related with the extremis mind-set, being part of it. Neuroticism was not identified as being a variable that could have a direct influence on mind-set extremism, or an indirect influence through personality. Religious adherence is a good predictor of extremist ideology. A global personality factor consisting in low Intellect/Imagination, low Extraversion and high Agreeableness seems to be a vulnerability factor that influences people to believe in extremist ideology.

## Introduction

There are different counter-radicalization initiatives and programs, and one critique of them is the lack of conceptual framework regarding the mechanisms of change (Horgan, 2016; Gøtzsche-Astrup, 2018). There is little evidence regarding risk factors for radicalization in the scientific literature (Monahan, 2012). Horgan (2016) pointed out that most of the research on radicalization, extremism and terrorism has been conducted by specialists in social science whereas just a few psychologists were involved in the field. He also mentioned that little is known about why people get radicalized and became terrorists. The present article tries to shed light on these ponderings as well as to emphasize possible psychological mechanisms underlying radicalization and extremism.

There seems to be no unanimous consensus regarding definition of radicalization and extremism. Sometimes terms such as radicalization, extremism, and terrorism overlap. However, the starting point for this research was the definition of radicalization offered by McCauley and Moskalenko (2008, p. 416): “Functionally, political radicalization is increased preparation for and commitment to intergroup conflict. Descriptively, radicalization means change in beliefs, feelings, and behaviors in directions that increasingly justify intergroup violence and demand sacrifice in defense of the in-group.” As stated by Borum (2011), radicalization refers to the process through which extremist beliefs and ideology develop, and violent extremism, terrorism are action pathways for the expression of extremist beliefs. There are both individual and group factors that influence people to renounce dialogue, agreement, and tolerance and then to use violent extremism (Schmid, 2013).

Various studies distinguished between radicalized individuals who commit violent acts within a radical group and individuals who act alone or as part of autonomous cells. Terrorists who come from the first category are psychological normal male adolescents or young adults on the mid-twenties (Silke, 2008). Based on risks factor described by Monahan (2012), a terrorist is usually male, aged between 20 and 29 years, unmarried or married to a spouse inside the terrorist organization. Their professional, educational and financial levels are similar to those of population from which they emerged. Terrorists are people generally without any clinical diagnosis of schizophrenia, bipolar disorder, and alcohol or drug abuse, personality disorders as psychopathy. Khosrokhavar (2014) described radicalized people in Europe as being younger, often teenagers and he surmised that the number of women involved was on the increase since 2010. Studies analyzing terrorists who acted alone or as part of autonomous cells characterized them as being mostly male, average age 30 years, most unemployed and well-educated (high school and college). Those who acted alone were never married and those who acted in autonomous cells were married (Meloy et al., 2015; Meloy and Gill, 2016). Unlike extremist individuals who committed violent acts within a radical group, the majority of those who acted alone had a mental disorder diagnosis and all who acted as part of the autonomous cells had a history of criminal violence beyond the terrorism.

Recent studies support the idea that there are many paths into radicalization, each of them being influenced by a variety of factors grouped into eight clusters: Affect/Emotion, Behaviors, Cognitive Style, Beliefs/Ideology, Attitudes, Social factors, Identities, and Capacities (Borum, 2011, 2015). Emotions such as anger, hate, and disgust can serve as activating motivational forces for violent behavior. All of them are linked with themes of perceived injustice and humiliation. As mentioned above, a history of violence was found only for terrorists who acted as part of the autonomous cells. The cognitive style is absolutistic and dogmatic, usually expressed in “black and white,” “I must be right thinking because I said so,” unrealistic expectancies that others want to threaten their self-esteem. Ideology is represented by a set of beliefs that guides and justifies violent acts, and these includes themes of good and evil, personal or group grievance often expressed in a narrative frame of reference. Social factors involved can be categorized in indicative of a support group that has pro-violence attitudes but also social alienation, with a mind-set that proclaims they are evil and we are good. Identity includes core beliefs by which an individual define himself and to which he attributes his worth. Finally, the person has to have the physical, intellectual and social capabilities to execute the extremist behaviors. Additionally, personal grievance, frame ideology and changes in thinking and emotions were revealed as risks factors by (Meloy and Gill, 2016) and Meloy et al. (2015).

Gøtzsche-Astrup (2018) reviewed the psychological mechanisms of radicalization that are empirically supported: (1) most extremists come from the ranks of normal population without a diagnosis of mental disorder; (2) when they are facing negative life events, all these people may experience uncertainty or epistemic vagueness and identify with one clear group rather than many; (3) emotions as anxiety and anger may be expressed by radicalized people facing life adversities through aggressive behaviors; (4) radicalized behaviors have motivational functions, by which individuals try to decrease emotional distress triggered by different life events and radicalized ideologies have a compensatory role (defensive role); (5) radicalized persons generally perceive a threat of their “sacred value” (6) there are small group dynamics that drive the process to behavioral extremes.

Following the pyramid model proposed by McCauley and Moskalenko (2008) and the staircase model described by Moghaddam (2005), the majority of people or members of the community represents the foundation of the pyramid or the basement floor. At this level, some psychological factors or mechanisms are acting to influence people to be vulnerable to radicalization. As the number of people associated with the higher levels of the pyramid decreases, their radicalization increases.

In the next sections, the models regarding radical belief system, irrational beliefs and personality traits were reviewed.

## Extremist Mind-set or Radical Belief System

Stankov et al. (2010a) proposed a tripartite model of militant extremist mind-set by looking for recurrent patterns of thinking, feeling and behaving described by historical, psychological and literacy texts, by texts written by authors from different militant extremist groups and by specific words and content categories used by extremist propaganda. The tripartite model includes: (1) Proviolence beliefs—violent acts are accepted, justified and advocated for use to get revenge or for the acceptance, justification, and even advocacy for the use of revenge and redemption; (2) Vile World—world is seen as being miserable, hopeless, irremediable, evil, one in which the modern governments are heading for destruction because they do not follow moral values by embracing materialistic approaches as free market; (3) Divine Power—beliefs in God's help, life after death, martyrdom and good intentions of their leaders.

Based on a linguistic analysis of the texts which described terrorist organizations and texts available on internet related with extremism, Stankov et al. (2010b) identified three components of the militant extremist mind-set: (1) War—justification of violent acts as war, armed struggle, terror, and willingness to kill people; (2) God—the name of God, Allah is connected with war, the acts of violence are seen as noble and moral because they are done in the name of God; (3) West—Western countries are seen as evil, immoral, committing violence, and aggression against other countries, so that violence against West is justified.

From the foregoing, the extremist mind set is conceptualized as a dimension that exists in general population and the extremism varies on a continuum from non-existent, very weak to very strong endorsement. People with a strong commitment to extremist mind-set are those that show extremist emotions and behaviors, and they have high probability to become terrorists.

Studying Islamic young people living in Netherland, Doosje et al. (2013) described a radical belief system comprised from four elements: (1) Perceived illegitimacy of authorities—mistrust of the authorities, which treated people in discriminatory and abusive ways; (2) Perceived in–group superiority—all other groups are perceived as worthless; (3) Perceived distance to other people who live differently—the in-group norms and values are in conflict with those of the dominant culture; (4) Perceived societal disconnectedness—the feeling that one does not belong to the mainstream society.

## Irrational Beliefs

The basic irrational belief that underlies human disturbance is the absolutistic “must” or demand statements about self, others and life conditions (Ellis, 1994). Demandingness is the tendency to transform wishes, desires, and preferences into absolutistic requirements. Following the level of cognition proposed by DiGiuseppe et al. (2014), demandingness is a core irrational belief which serves as a basic life philosophy.

Furthermore, Ellis differentiated between two important irrational beliefs: low frustration tolerance (LFT) and global evaluation of human worth. At the beginning of 1980s, Albert Ellis introduced a new concept “discomfort anxiety,” which he contrasted with “ego anxiety” (Ellis, 1979, 1980). He defined discomfort anxiety as “emotional tension that resulted when people felt (1) that their comfort (or life) was threatened, (2) that they should or must get what they want (and should not or must not get what they do not want), and (3) that it was awful or catastrophic (rather than merely inconvenient or disadvantageous) when they do not get what they supposedly must get” (Ellis, 2003, p. 183). Ego anxiety was defined as “emotional tension that results when people feel (1) that their self or personal worth is threatened, (2) that they should or must perform well and/or be approved by others, and (3) that it is awful or catastrophic when they don't perform well and/or are not approved by others as they supposedly should or must be” (Ellis, 2003, p. 183).

DiGiuseppe et al. (2014) proposed four categories of irrational beliefs: demandingness as core belief, respectively LFT, global evaluation of human worth and awfulizing as logical derivatives of demandingness. Five possible content areas were revealed: affiliation, achievement, comfort, fairness, and control. David et al. (2005) and Turner (2016) argued for primary evaluative belief or primary appraisal (demandingness) and three secondary appraisal processes or evaluative beliefs (LFT, global evaluation of human worth and awfulizing). Szentagotai et al. (2005) conceptualized demandingness and global evaluation of human worth as evaluative schemas; low frustration tolerance and awfulizing were described as appraisals (evaluative cognitions).

There were just a few studies found in the literature that offered empirical support for the absolutistic demands for fairness as a starting point of cognitive openness to radicalization (Moghaddam, 2005; Doosje et al., 2013).

Ellis considered LFT as probably the first irrational beliefs that young children develop (McMahon and Vernon, 2010). Low frustration tolerance (LFT) reflects perceived inability to withstand reality when it is not as one wants it to be—easy, pleasurable and comfortable. It is expressed in terms as “I cannot bear it, stand it, or face it.” Many studies described LFT as a multidimensional construct (Dryden, 1999; Harrington, 2005; Zvolensky et al., 2010; Bardeen et al., 2013; Bebane et al., 2015). Among its components were listed emotion intolerance, behavior intolerance, discomfort intolerance, effort intolerance, rules intolerance, entitlement (intolerance of unfairness and frustrated gratification), achievement intolerance (intolerance of frustrated achievement goals), uncertainty intolerance, ambiguity intolerance, work intolerance, etc. Feelings of uncertainty, self-uncertainty, and life-uncertainty are concepts related with radicalization and extremism. Adherents are motivated to reduce self-uncertainty by identifying with extremist groups that prescribed prototype behaviors and when they face personal uncertainty they engage in defensive compensatory acts as extreme conviction and idealistic approach (Doosje et al., 2013; Hogg and Adelman, 2013; McGregor et al., 2013; Hogg and Wagoner, 2017).

A very distinctive feature of rational emotive behavioral therapy is the theory of person worth. People have an innate tendency, but also learn to evaluate themselves globally. Parents teach children global evaluation of self and others. First they model global evaluation of others by their messages: “You must do things I am telling you to do and you are not good if you don't obey me!.” Secondly, the innate tendency of people to transform goals into necessities helps kids to take parental standards and turn them into absolutistic musts and then to evaluate themselves as being good or bad depending on their performance. In this way, people practice other and self/global evaluation over the years, rating themselves and others as good, valuable or bad, rotten depending on their success or failure in different tasks and life events. People forget that any person is in a process of becoming, that development is a dynamic process, and therefore people's worth cannot be really measured. Global evaluation of human worth cannot be empirically validated or falsified (Ellis, 1994). To do so would mean to evaluate or measure against some benchmark all of a subject's previously played roles in life, his or her present roles, and roles aspired to and yet undertaken and even unknown to a person. Ellis argued for evaluation of roles for sure, but not the evaluation of persons and he judged global self or other evaluation (above citation) as cornerstone for bigotry.

There are empirical studies supporting that extreme conviction and idealistic approach are used as a defense mechanism when people face different threats of self. Lüders et al. (2016) talked about self-concept uncertainty and conceptualized it as epistemic vagueness, the need for meaning and epistemic equilibrium. Jonas et al. (2014) postulated that people feel uncertainty anxiety and try to escape from it by using reactive defense mechanisms (the extremist mind set) when their epistemic equilibrium is shaky, when they have an insecure self-esteem and when their self-control is threatened. McGregor et al. (2013) found that extreme religious beliefs were determined by personal uncertainty through active achievement and relationship goals threatening. In another study, McGregor et al. (2005) empirically supported associations between insecure forms of high self-esteem (high explicit and low implicit self-esteem), concluding that self-esteem can be a sign of defensiveness and may result from repeatedly hiding implicit self-doubts with the display of explicit self-worth masks. These self-worth masks could be pride, avoidant or arrogant attachment style, narcissism and entitlement.

Doosje et al. (2013) revealed different psychological mechanisms that follow different paths to create a radical beliefs system. One path indicated that facing deprivation as a group member, someone may believe that his group's morals, values, standards, beliefs, and attitudes are more correct then those of out-group. This symbolic threat can lead to cognitive evaluation of in-group as being superior to out-groups (global evaluation of human worth), a belief that supports violent attitudes. Another path to violent attitudes is generated by expectancies that outgroup members will behave in ways that are a threat to the very existence of the in-group (realistic threat), followed by a great distancing toward people of the out-group.

Extremist leaders address this irrational beliefs of humans, by claiming for the existence of an absolute ideal world and for re-education or elimination of all people that ignore this guiding principle. Thus, decisions then are required to determine which people will live or not in order to enhance this principle. Ellis (1994) offered some suggestions regarding the logic behind such idealism and decision making. If a person thinks that he/she is not of worth, the corollary is that he/she thinks that life is not worth living. The only way a man can experience life is through him, he is the only channel to life. If life is worthwhile, it means the man is of worth. However, when people experience that epistemic vagueness, and they believe are nothing or weak people, they do not follow this logic way of thinking. Getting in contact with extremist ideology of martyrdom, people may think that life after death is worthwhile than their actual life. But there is no proof for that, because “no live person has ever really been dead and no dead person has ever returned to compare the life and death.” (Ellis, 1994, p. 201). Of course, in their process of radicalization, people ignore the empirical evidences. If individuals think that other people are not of worth, they think their lives are not worth living. The out-group members, who don't share the same morals, values, standards, beliefs, and attitudes are not of worth people, so their lives are not worth living. But all people are instruments, channels to life and if life is worthwhile, a logic conclusion will be that all channels are of worth. Thus, it is logically wrong to conclude that only some people are of worth.

## Personality Factors and Extremis

Referring to personality factors, Monahan (2012) stated that some research tried to distinguish terrorists from non-terrorists, but in the recent period the topic has been quite neglected, because the existing personality tests were unable to differentiate the individuals who would engage in extremist acts from those who wouldn't. For example, Bell et al. (2012) examined the relation between political attitudes and personality factors. Contrary to general expectations, the authors found no significant correlations between the variables. They observed that people scoring higher in the general factor of personality were more likely to take an interest in politics.

For the general population, Alizadeh et al. (2017) screened over 355,000 Tweeter messages of followers of non-violent organizations, comparing them with random users, followers of apolitical celebrities. Extremist followers were found to be less agreeable, less conscientious, less neurotic, and more open than non-extremists, but similar in extraversion. The authors concluded that personality might be more related with militancy than with the ideological orientation of the individuals. Also, it was noted that a profile of personality characterized by a low level of agreeableness and a high level of openness could form the foundations for political radicalization, because such individuals are insusceptible to the opinion of the others, but quick to spread their own.

In another study, Fauset (2014) aimed to investigate if undergraduate students supported extreme statements and to identify the relation between possible support and personality variables. The results showed a negative association between neuroticism, openness and agreeableness on one hand and believing that the acts of aggression are the solution for salvation (Proviolence), on the other. Agreeableness also correlated negatively with considering that the world is mean and miserable (Mean World). Openness was negatively associated with believing that God has a divine plan for humankind and that plan should be trusted. The extremist portrait described by Fauset suggested a rigid individual, prepared to use violence in order to affirm his opinion, and this originates in the belief that something is wrong with the world.

Concerning triple vulnerability theory, Barlow et al. (2014) emphasized the role of neuroticism in emotional disturbances. The authors proposed a triadic model of vulnerabilities: a general biological (heritable) vulnerability, a general psychological vulnerability and specific psychological vulnerability. Genetic and neurobiological factors represent the general biological vulnerability. The general psychological vulnerability consists in a common sense of unpredictability and uncontrollability associated with a perceived inability to cope with life events. The interaction between these two factors generates neuroticism, defined as “the tendency to experience frequent, intense negative emotions associated with a sense uncontrollability in response to stress” (Barlow et al., 2014, p.481). A high level of neuroticism associated with specific psychological factors explains particular emotional disorders.

In their literature review, Campelo et al. (2018) pointed out that radicalization cannot be directly linked to mental illness, but some personality traits can be so associated: anti-social, obsessive, and histrionic traits. Intense depressive emotions were often reported among radicalized youth, without meeting the criteria for a major depressive episode. Suicidal ideation could be identified especially for martyrdom cases. A history of addictive behavior, risky behavior, and sensation seeking was also identified. Early experiences of abandonment were present in the radicalized youth life trajectories. Bhui et al. (2014) found that depression was more specific to people who showed sympathy toward violent protests and to terrorism.

Stankov et al. (2010b) concluded that not all terrorist acts are committed by individuals who suffer from a mental disorder, but more likely some of them shared a particular configuration of personality traits. They found moderate negative correlations between consciousness, agreeableness and extraversion on one hand and believing that the acts of aggression are the solution for salvation (Proviolence) on the other hand. This is in accordance with their conclusions, emphasizing that a common personality matrix was unlikely.

Summing all these, we assume that irrational beliefs and a constellation of personality factors could be two possible psychological mechanisms that put people in a vulnerable spot and might influence them to develop a radical belief system or an extremist mind-set. More specifically, we hypothesize that LFT beliefs, global evaluation of human worth, personality traits (neuroticism, agreeableness, intellect imagination, and conscientiousness) predicts the extremist mind-set.

## Methods

### Participants

The sample consisted in 295 Romanian adolescents, aged between 15 and 18 years, the mean age being 16.41 (*SD* = 0.936). There were 125 girls (42.4%) and 170 boys (57.6%). In terms of ethnicity, 176 were Romanians (59.7%), 70 of them Hungarians (23.7%), and 49 were Roma adolescents (16.6%). Concerning religious denominational affiliation, 172 adolescents belonged to Orthodox Christian religion (58.3%), 23 of them to Roman Catholic Church (7.8%), 61 to Evangelical Christian Church (20.7%), and 39 to Reformed denominations (13.2%). All participants were high school or vocational school students from the western part of Romania.

### Measures

*Militant Extremist Mind-Set Scale* was developed by Stankov et al. (2010b) for the measurement of militant extremist mind-set. Factor analysis of militant extremist statements produced 3 dimensions: (a) justification and advocacy of violence (War factor), (b) violence in the name of God (God factor), and (c) blaming Western nations for the problems in the world today (West factor). Each statement was accompanied by a five point Likert scale. Cronbach's alphas ranged from 0.80 to 0.86 (War 0.81, God 0.86, West 0.80).

*Children and Adolescent Scale of Irrationality* (CASI, Bernard and Cronan, 1999, adapted for Romanian population by Trip, 2007) was used to measure irrational beliefs. The scale contains 28 items measuring four categories of irrational beliefs: Global evaluation of self, LFT to general rules, LFT to work and Demands for fairness. Participants expressed their agreement or disagreement using a 5–point Likert scale from 1 to 5 (1 = strongly disagree, 2 = disagree, 3 = not sure, 4 = agree, 5 = strongly agree). Cronbach's alpha coefficients obtained for Romanian population (Trip, 2007) were 0.85 for LFT to Rules, 0.74 for Global evaluation of self, 0.62 for Demands for fairness and 0.65 for the LFT to Work scale. Similar values of Cronbach's alpha coefficients were found for the present sample: 0.79 for LFT to Rules, 0.79 for Global evaluation of self, 0.41 for Demands for fairness and 0.61 for the LFT to Work.

*Mini-IPIP Scale* was developed by Donnellan et al. (2006) as a short-form for the International Personality Item Pool (Five-Factor Model) proposed by Goldberg (1999). It contains 20 items with four items per factor and measures the five dimensions of the Big Five Model (Extraversion, Agreeableness, Conscientiousness, Neuroticism, and Intellect/Imagination). Each item is a phrase describing a behavior; participants' task is to indicate how accurate this phrase is for them using a five Likert scale. In contrast with scores obtained by Donnellan et al. (2006) the values of Cronbach's alphas recorded for this sample were lower for all subscales, and ranged from 0.57 to 0.76 (Extraversion 0.76, Agreeableness 0.67, Conscientiousness 0.68, Neuroticism 0.49, Intellect/Imagination 0.57).

### Procedure

The present study was conducted in several schools from Bihor County located in the western part of Romania. Adolescents took part on a voluntary basis in the study after obtaining the written, informed consent from their parents. The questionnaires mentioned above were administered in a paper-pencil format to 400 students, between October 2017 and March 2018. Only 295 (73.75%) of them were used due to missing answers or protocols not being returned to researchers.

## Results

Firstly, we conducted a confirmatory factor analysis (CFA) on structure of Militant Extremist Mind-Set Scale using AMOS 23. We hypothesized a three-factor model of the extremist mind-set to be confirmed in the measurement portion of the model. We evaluated the assumptions of multivariate normality and linearity through SPSS 23.0. Using box plots and Mahalanobis distance, we observed 38 univariate or multivariate outliers and we removed them.

The final sample size was 257 and there were no missing data. Goodness of fit indices included the chi-square (χ^2^), the comparative fit index (CFI), Tucker-Lewis Index (TLI), and the root-mean-square error of approximation (RMSEA). Schreiber et al. (2006) suggested the following cutoff levels for determining model fit: CFI and TLI ≥0.95, RMSEA <0.06 to 0.08.

**Figure 1:**
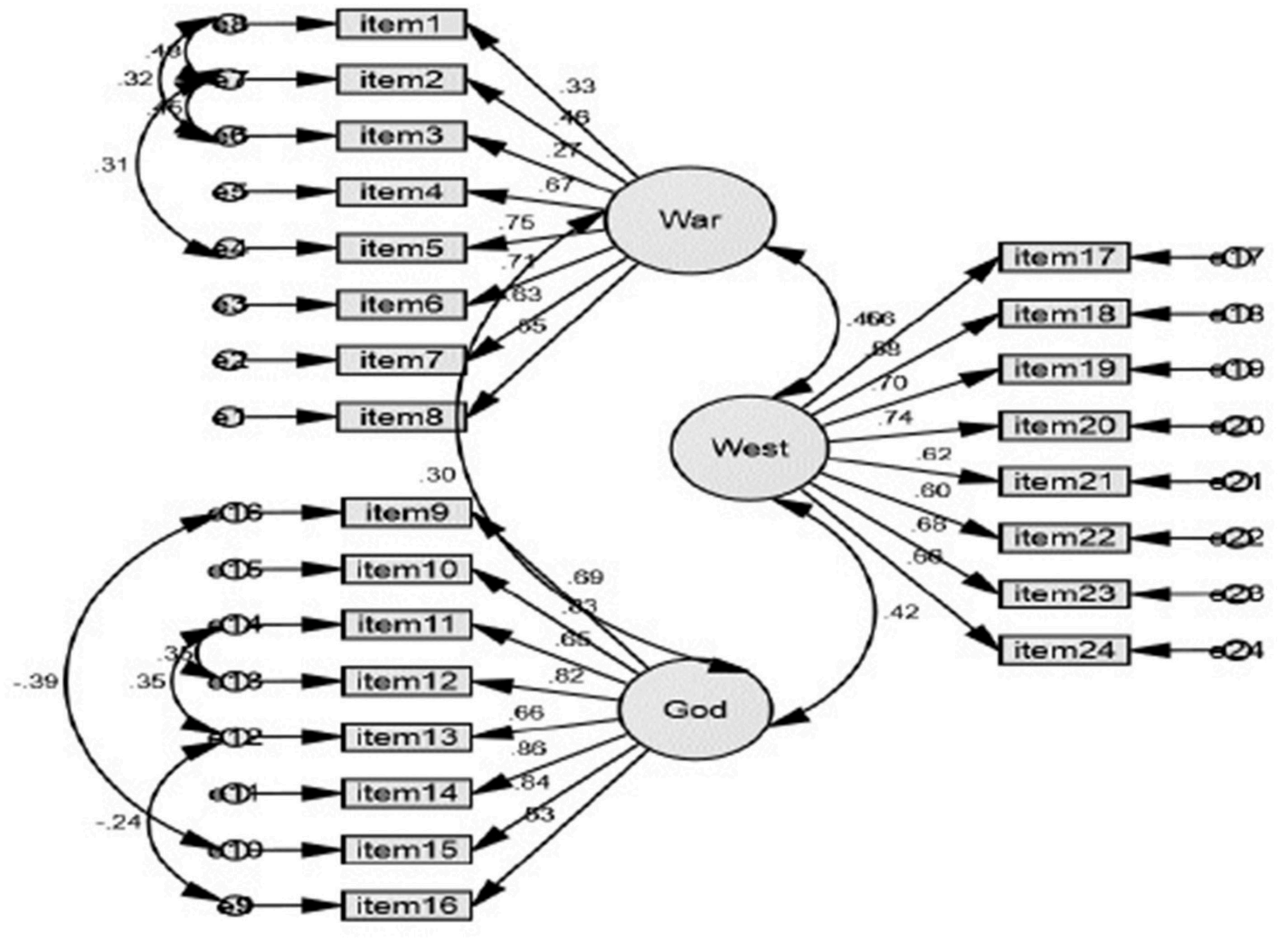
Confirmatory factor analysis militant extremist mind-set scale.

Our initial results based on CFA indicated an adequate model fit of the tested models corresponding to the factor structure of extremist mind-set. The comparative fit index (CFI) = 0.90, the Tucker-Lewis fit index (TLI) = 0.89, and the RMSEA = 0.06, χ^2^*(df* = 241*)* = 499.110, *p* < 0.01. Standardized parameter estimates are provided in Figure 1. Similar with Stankov et al. (2010b), the first eight items are loaded on War factor (justification and advocacy of violence), the next eight items are loaded on God factor (violence in the name of God), and the last eight items are loaded on West factor (blaming Western nations for the problems in the world today). The observed standardized factor loadings exceeded Hair et al. (1998) strict cutoff criteria of 0.60 for a number of 16 items out of 24 and exhibited statistically significant factor loadings above 0.40 for 6 out of 24 items. There are two exceptions for item 1 and 3, the observed standardized factor loadings are below 0.40, but we did not excluded them because the theoretically assumed relationship between them and the “War” factor (War is not a path to salvation; There is no justification for terrorism of any kind). All three subscales can be reliably used for the assessment of militant extremism, the values of Cronbach's alphas are 0.79 and higher (War 0.79, God 0.90, West 0.84).

Both standardized and unstandardized estimates are shown in Table 1.

**Table 1:** Unstandardized and standardized coefficients confirmatory analysis extremist mind-set scale.

			**β**	***B***	***SE***
Item 8	<---	War	0.698	0.547	0.091
Item 7	<---	War	0.820	0.629	0.094
Item 6	<---	War	1.000	0.708	–
Item 5	<---	War	1.001	0.747	0.101
Item 4	<---	War	0.953	0.667	0.104
Item 3	<---	War	0.423	0.265	0.111
Item 2	<---	War	0.641	0.463	0.100
Item 1	<---	War	0.508	0.333	0.107
Item 16	<---	God	0.529	0.526	0.061
Item 15	<---	God	0.877	0.838	0.055
Item 14	<---	God	0.942	0.860	0.056
Item 13	<---	God	0.775	0.661	0.067
Item 12	<---	God	1.000	0.823	–
Item 11	<---	God	0.787	0.654	0.058
Item 10	<---	God	1.010	0.831	0.064
Item 9	<---	God	0.825	0.690	0.069
Item 17	<---	West	0.762	0.561	0.090
Item 18	<---	West	0.777	0.584	0.089
Item 19	<---	West	0.964	0.697	0.092
Item 20	<---	West	1.000	0.742	
Item 21	<---	West	0.910	0.618	0.098
Item 22	<---	West	0.821	0.596	0.092
Item 23	<---	West	0.916	0.679	0.090
Item 24	<---	West	0.853	0.665	0.085

Two confirmatory factor analyses were also conducted for the other two scales used in this study, CASI and Mini-IPIP. The results confirm the previous models for both scales, including items loading on factors. The comparative fit index (CFI) = 0.81, the Tucker-Lewis fit index (TLI) = 0.80, and the RMSEA = 0.07, χ^2^ *(df* = 343*)* = 841.110, *p* < 0.01 indicate an acceptable fit between the model and the observed data collected by CASI. The values recorded for Mini-IPIP support a better fit: *CFI* = 0.88, *TLI* = 0.79, RMSEA = 0.06, χ^2^ *(df* = 16*)* = 347.294, *p* < 0.01.

In accord with the cutoff levels suggested by Schreiber et al. (2006), the χ^2^ to df. ratio (χ^2^ to df ≤ 2 or 3) and RMSEA (<0.06–0.08) results indicated a good model fit of the factor structure of all three measurements. The Comparative Fit Index (CFI) and Tucker Lewis Index (TLI) are measures of how much better the model fits the data compared to a baseline model where all variables are uncorrelated. The obtained values did not satisfied the required model fit statistics. For the Extremist Mind-Set Scale, the CFI value of 0.90 indicated a reasonable fit (Hu and Bentler, 1999), but TLI was below this cutoff. In the case of CASI and Mini-IPIP, both CFI and TLI values are below 0.90. A possible explanation for these results could be related to the statistically significant associations between latent factors of each of the three measurements. For example, the association between factor West and factor God is moderate (*r* = 0.51) and the correlations between factor War with both factors West (*r* = 0.46) and God (*r* = 0.39) is weak to moderate. Almost moderate associations were found between some of Mini-IPIP factors: Agreeableness—Intellect/ Imagination (*r* = −0.46), respectively Agreeableness—Extraversion (*r* = −0.45). Weak correlations but significant were found between the other factors too (see Table 2). With regard to CASI, our results support the explanation offered by Hyland et al. (2014) related to the contamination of the process factors (demandingness, LFT, global evaluation, etc.) by contextual factors (work, rules, and fairness). Table 2 reports significant strong correlations between three of the latent factors of CASI, all measuring LFT process: LFT work—Demand for fairness (*r* = 0.84), LFT work—LFT rules (*r* = 0.70).

**Table 2:** The means, standard deviations, and inter-correlations of all constructs.

**Observed variables**	***M***	***SD***	**2**	**3**	**4**	**5**	**6**	**7**	**8**	**9**	**10**	**11**	**12**
1. West	2.90	0.69	0.511^**^	0.467^**^	−0.139^*^	−0.171^**^	0.316^**^	−0.052	0.121^*^	−0.171^**^	0.082	0.042	−0.129^*^
2. God	2.96	1.15	–	0.399^**^	−0.019	−0.026	0.174^**^	−0.012	0.000	−0.122^*^	0.073	−0.107^*^	0.045
3. War	1.93	0.62	–	–	−0.06	−0.177^**^	0.297^**^	0.106^*^	0.110^*^	−0.194^**^	0.217^**^	0.040	−0.080
4. LFT work	3.20	0.61	–	–	–	0.848^**^	−0.012	0.709^**^	−0.084	0.126^*^	−0.029	0.182^**^	0.285^**^
5. Demandingness	2.39	0.47	–	–	–	–	−0.167^**^	0.247^**^	−0.086	0.176^**^	−0.061	0.062	0.160^**^
6. Global evaluation	0.75	0.26	–	–	–	–	–	0.186^**^	0.360^**^	−0.139^*^	0.015	0.233^**^	−0.103
7. LFT rules	2.01	0.58	–	–	–	–	–	–	−0.051	0.045	0.000	0.249^**^	0.342^**^
8. Neuroticism	0.58	0.36	–	–	–	–	–	–	–	−0.128^*^	−0.029	0.240^**^	−0.339^**^
9. Intellect/Imagination	0.04	0.58	–	–	–	–	–	–	–	–	−0.469^**^	−0.049	0.366^**^
10. Agreeableness	0.21	0.67	–	–	–	–	–	–	–	–	–	0.075	−0.453^**^
11. Conscientiousness	0.17	0.82	–	–	–	–	–	–	–	–	–	–	−0.004
12. Extraversion	0.94	0.74	–	–	–	–	–	–	–	–	–	–	–

** *p < 0.01*,

* *p < 0.05, 1 = West, 2 = God, 3 = War, 4 = LFT work, 5 = Demandingness, 6 = Global Evaluation, 7 = LFT rules, 8 = Neuroticism, 9 = Intellect / Imagination, 10 = Agreeableness, 11 = Conscientiousness, 12 = Extraversion*.

The results tend to demonstrate that our sample supports mostly alternatives of 1 (strongly and completely disagree) or 2 (moderately or mostly disagree) of the Likert scale for the War statements. These indicate that the sample disagrees with the use of violence. For the other two factors, the participants offered more answers of 3 (*neither agree nor disagree)*, 4 (*moderately or mostly agree*), and 5 (*strongly and completely agree*). The means reveal that the sample neither agree nor disagree with violence in the name of God and with the blame of Western nations for the problems in the world today. For both subscales, participants are closer to being undecided.

Regarding irrational beliefs, and in reference to Romanian population standards of CASI, the sample recorded high scores for LFT to rules and demands for fairness. A medium level result eventuated for Global evaluation of self and a very low level for LFT to work. Concerning personality dimensions, the sample was characterized by a medium level of Extraversion, Agreeableness, Conscientiousness, Neuroticism, and Intellect/Imagination.

Further on, based on data from 257 participants, we performed farther SEM analysis with AMOS 23 statistical package. We evaluated the assumptions of multivariate normality and linearity and observed 15 multivariate outliers (*p* < 0.001). We removed the 15 outliers from the subsequent analyses, leaving a final sample size of 242 (257 minus 15); there were no missing data. We chose maximum likelihood parameter estimation over other estimation methods because the data were distributed normally (Kline, 2011).

We establish three latent variables: extremism, personality and irrational beliefs, representing them by circles. The measure variables for extremism were the tree factors revealed by confirmatory analysis: War, God, and West. The five dimensions Extraversion, Agreeableness, Conscientiousness, Neuroticism, and Intellect/Imagination represented the measure variable for personality. Four measure variables were identified for irrational beliefs: Global evaluation of self, LFT to rules, LFT to work and Demands for fairness. Rectangles represent measure variables. Based on confirmatory analysis, the input scores were calculated for each subscale and these scores were used in the analyses that followed. The regression coefficient has been fixed to 1 for the first factor that loaded on each scale as it was confirmed by literature (War-Extremism, LFT rules—Irrational beliefs, Extraversion—Personality) and for each error. Religion is a categorical observed variable, the affiliation to Orthodoxy being coded with 1 and all other affiliations were coded with 0. Categorical observed variable were also gender (1–male, 0–female) and ethnicity (1–Romanians, 0–others).

We specified fifth structural models. Goodness of fit indices included the comparative fit index (CFI), Tucker-Lewis Index (TLI), and the root-mean-square error of approximation (RMSEA), and a fit of >0.95 for the CFI and TLI and RMSEA < 0.06 was judged to indicate adequate fit. Also reported are chi-square, goodness of fit test and the standardized root-mean-square residual (Schreiber et al., 2006).

Model fit statistics are summarized for all five models in Table 3.

**Table 3:** Fit statistics from five models.

**Model**	***χ^2^***	***df***	***p***	**CFI**	**TLI**	**RMSEA**	**SRMR**
Model 1	474.00	88	0.000	0.77	0.72	0.13	0.13
Model 2	269.014	63	0.000	0.86	0.83	0.11	0.11
Model 3	79.907	24	0.000	0.85	0.78	0.09	0.08
Model 4	37.19	19	0.008	0.94	0.91	0.06	0.05
Model 5	32.465	19	0.028	0.95	0.93	0.05	0.04

The first model which indicated irrational beliefs, personality, gender, religion and ethnicity as directly influencing extremist mind-set, fit poorly (RMSEA = 0.11). Estimates of regression weights showed that irrational beliefs, gender and ethnicity do not have a direct effect on extremist mind-set. Global evaluation of self seems not to be part of the latent variable named irrational beliefs. Conscientiousness was not an important dimension of personality as a latent variable having a direct effect on extremism.

The second model was created by eliminating gender and ethnicity, keeping irrational beliefs and personality as latent variables and adding global evaluation and conscientiousness as observed variables with direct effect on extremism. The fit was poor. The third model that allowed direct effect of global evaluation of self (observed variable), personality (latent variable) and religion (observed variable) fit poorly (RMSEA = 0.09). Estimates of regression weights do not support neuroticism as significant component of personality as a latent variable that directly influenced extremism. The fourth model kept personality, global evaluation of self and religion as variables directly influencing the extremism. The model showed a good fit, but CFI and TLI are lower than desired value for adequate fit.

**Figure 2:**
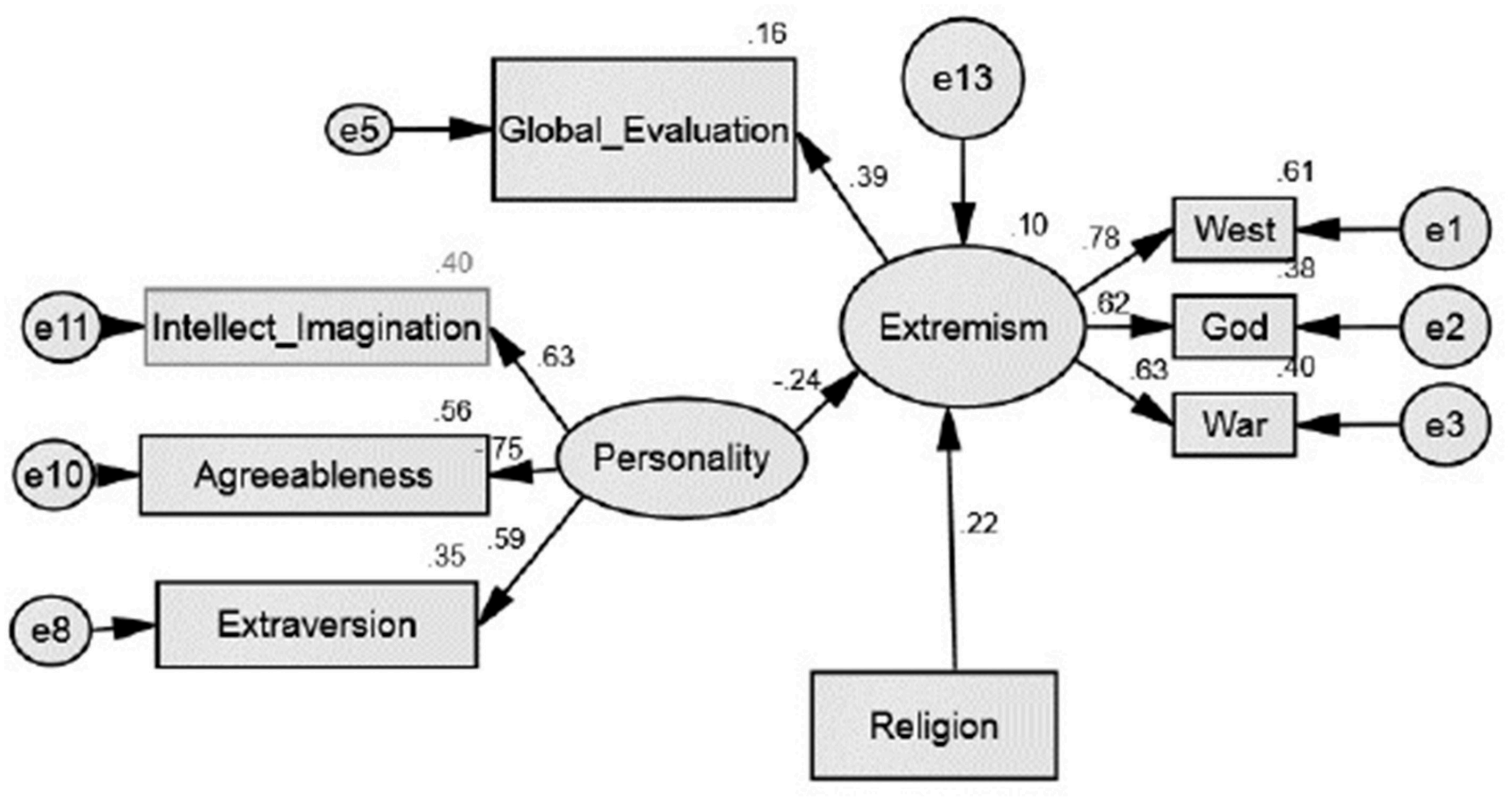
Structural equation model.

The fifth model had adequate fit based on all three fit indices including the RMSEA (0.054), CFI (0.958), and SRMS (0.047). The value of TLI (0.937) is still lower than 0.95. This model exclude neuroticism and kept global evaluation of self as part of the extremist mind-set. The model supports personality as a variable that has a direct effect on extremist mind-set (Table 4). A constellation of personality traits composed by high intelligence and imagination, high extraversion and low agreeability seems to be negatively related with extremist mind-set. Global evaluation of self seems to be a salient component of the extremist mind-set. The model is represented in Figure 2.

**Table 4:** Results from structural equation modeling.

	**β**		***B***		***SE***		***R^**2**^***	
	**Personality**	**Religion**	**Personality**	**Religion**	**Personality**	**Religion**	**Personality**	**Religion**
**DIRECT EFFECT**								
Extremism	−0.21	0.17	−0.24	0.13	0.08	0.05	−0.21	0.17
**INDIRECT EFFECT**								
War	−0.21	0.17	−0.15	0.13				
God	−0.38	0.30	−0.14	0.13				
West	−0.29	0.23	−0.18	0.16				
Global evaluation	−0.05	0.04	−0.09	0.08				
**TOTAL EFFECT**								
Extremism	−0.21	0.17	−0.21	0.21				
War	−0.21	0.17	−0.15	0.21				
God	−0.38	0.30	−0.14	0.13				
West	−0.29	0.23	−0.18	0.13				
Global evaluation	−0.05	0.04	−0.09	0.10				

## Discussions

Results in this study do not support the hypothesis that irrational beliefs are psychological mechanisms that make people vulnerable to extremism. The models show that LFT to work, LFT to rules and Demand for fairness are grouped under the irrational beliefs latent variable. Global evaluation of self seems to stand out independently. By analyzing the content of irrational beliefs, it can be observed that the items measuring the three cognitions LFT to work, LFT to rules and Demand for fairness refer to academic domain. The only subscale that contains general items without referring to a specific domain is Global evaluation of self. It seems that there is a domain dissociation, the results obtained by using measures focused on academic domain cannot be extrapolated to political conviction aria. Extremist political beliefs cannot be predicted based on irrational beliefs (LFT, Demandingness for fairness) expressed in academic context. Rather, it could be helpful to measure irrational beliefs expressed in political domain or at least to keep the items in a general expressed form, without making references to a specific area. Both scientific research and newspapers articles based on declarations of perpetrators' families enable arguments for domain dissociation. For example, low levels of educational and socio-economic backgrounds were not found to be characteristic of terrorists (Krueger and Maleckova, 2003).

As example, perpetrators of Barcelona attacks in 2017 were described by others as people well integrated in society.

Doosje et al. (2013) and Moghaddam (2005) identified absolutistic demands for fairness as the starting point of the cognitive openness to radicalization. The individual thinks that his group does not have the same advantages as other groups, which is not fair or just. Our results support the idea that we cannot predict radical beliefs and behaviors based on demand for fairness cognitions expressed in academic area. It seems that beliefs with academic content (“It is not fair when teachers evaluate us in a wrong way, they always be correct”) does not have the same power in predicting radical ideology and behaviors as political beliefs (“It is not fair that Hungarians does not have territorial autonomy in Transylvania”) or as general beliefs (“People should always behave in a correct way, if not this is unfair”). But these conjectures are just a suppositions; not presently supported by empirical evidences.

The adequate fit model includes Global evaluation of self in the extremist belief system. This is similar to model of radical belief system described by Doosje et al. (2013). Perceived in–group superiority and perceived out-groups worthless along with perceived distance to other people who live differently as the in-group norms and values being in conflict with those of the dominant culture are different forms to express global evaluation of human worth belief. Uncertainty—identity theory postulates that when self-uncertainty became chronic, people are strongly attracted by extremist groups (Hogg and Wagoner, 2017).

When people face different threats of self, they experience raised anxious uncertainty as a consequence of behavioral inhibition system activity. They try to escape from it by using reactive defensive strategies as extreme conviction and idealistic approach (McGregor et al., 2013; Jonas et al., 2014; Lüders et al., 2016). In this study, not measured were any emotional variable, so future studies could be focused on the relationship between Global evaluation of self, LFT to uncertainty and anxiety. Regarding LFT beliefs, this study included two forms, LFT to work and LFT to rules, both with an academic content. In future research it could be interesting to assess the influence of LFT to uncertainty in relationship with Global evaluation of human worth and anxiety on extremist belief system.

CASI is currently the most powerful and important tool for measuring children and adolescents' irrational beliefs. However, CFA results showed that its factorial model did not satisfied the required model fit statistics. This was mainly due to the confusion of the processes with the contexts of irrational beliefs. CASI measures three processes of irrational beliefs demandingness, LFT and global evaluation of self, but the contents of items are related with academic work, rules and fairness. Thus, CASI's original structure consists in four confounded factors: global evaluation of self, LFT to general rules, LFT to work and demands for fairness. A strong correlation between the last three factors was revealed in this study. These construct validity problems of CASI could be another explanation for our SEM results. The three factors of LFT to general rules, LFT to work and demands for fairness loaded on the latent variable named irrational beliefs. Global evaluation of self has behaved as an independent factor that was not part of this latent variable. In addition, CASI does not assess global evaluation of others, an irrational belief important in the radicalization process claimed in previous studies (Doosje et al., 2013). For a better estimate of the relationship between irrational beliefs and extremism in adolescence, future studies could use an improved form of CASI or could develop a new measurement that includes global evaluation of others too.

Religion affiliation is a predictor of extremist mind-set. In the current study, the affiliation to Christian Orthodoxy increased the chance to endorse beliefs that support violence in the name of God, and to blame Western nations for the problems in the world today. Such beliefs justify and advocate violence, and also include a generalized evaluation and denigration of self. Domain dissociation is evident again. Christian philosophy postulates that man is made in the image and likeness of God. Therefore, man having the image and likeness of God cannot be worthless. Jesus, it could be argued, was a model of man who practiced unconditional self and other acceptance. The distinction between acceptance of sinner, but not of the sin is obvious. Even though a person is a Christian and believes in Christian philosophy, he/she still can globally judge himself/herself or others, thinking that a person (self or other) can be worthless.

All models that included Neuroticism as an observed variable measuring personality as latent variable did not meet adequate fit. A possible limit for these results might be the fact that the subscale of Neuroticism (Mini-IPIP Scale) does not have a very good reliability. The model that shows good fit does not include neuroticism as a variable that could have a direct influence on extremist mind-set or an indirect influence through personality. The study conducted by Fauset (2014) found neuroticism to be negatively related with pro-violence beliefs. In their study, Stankov et al. (2010a) obtained positive correlations between pro-violence beliefs and neuroticism. The belief that the present-day world is corrupt (Vile World) was also positively correlated with psychoticism. Neuroticism is defined by Barlow et al. (2014) as propensity toward experiencing more frequent and intense negative emotions in life situations perceived as uncontrollable. According to triple vulnerability theory, neuroticism is a general psychological factor of vulnerability to mental illness. Recent reviews (Campelo et al., 2018) concluded that radicalization cannot be directly linked to mental illness. More research is needed in order to test the validity of triple vulnerability theory in the case of radicalization and extremism.

Stankov et al. (2010a) found negative correlations between extremist mind-set and Extraversion, Agreeableness, Conscientiousness and Intellect/Imagination. Their subscale Divine Power (violence can be justified if it is committed in the name of God) positively correlated with Conservatism. The research done in the area of personality and politics revealed negative correlations between Intellect/Imagination and conservative or right-wing orientations (Caprara et al., 2006; Alford and Hibbing, 2007), but not obtained consistent results for the other personality dimensions. Eysenck (1954) postulated that a low level of intelligence was associated with a tendency to extreme political attitudes, idea supported also by Rindermann et al. (2012).

In recent years, many studies offer empirical support for the existence of a single global personality factor that can be predicted by stable behaviors manifested in adolescence and that is positively correlated with Extraversion, Intelect/Imagination, and Conscientiousness and negatively related with Neuroticism (Loehlin, 2012; Dunkel et al., 2014). Bell et al. (2012) concluded that global personality factor helps people to avoid political extremes and keep political moderation. The adequate model in this study identified a possible global personality factor that negatively influence extremist mind-set. A pattern of personality traits expressed through low Intellect/Imagination, low Extraversion and high Agreeableness seems to make people vulnerable to extremist ideology. The opposed pattern consisting in high Intellect/Imagination, high Extraversion and low Agreeableness could be a protective factor.

To conclude, global evaluation of self seems to be the only irrational belief that is somewhat related with the extremist mind-set by being part of it. In the statistically adequate model in the current study, neuroticism was not identified as being a variable that could have a direct influence on extremist mind-set or an indirect influence through personality. Religion is a good predictor of extremist ideology. A global personality factor consisting in low Intellect/Imagination, low Extraversion and high Agreeableness seems to be a vulnerability factor that inflicts people to believe in extremist ideology. From what we know, this is the first study that empirically investigates the relationship between irrational beliefs and extremist mind-set and our results need to be replicated in future studies for more accurate conclusions. The current study was conducted within a sample of adolescents without extremist or terrorist behaviors. In order to develop a most robust picture of the cognitive factors involved in radicalization, future research should preferably retest the model among extremist and radicalized adolescents groups.

## Ethics Statement

This study was carried out in accordance with the recommendations of Code of Ethics of University of Oradea. The protocol was approved by the Ethics Committee for Research, Faculty of Socio-Humanistic Sciences, University of Oradea. In accordance with the Declaration of Helsinki, all parents gave written informed consent for adolescents' participation in the study.

## Author Contributions

All authors listed have made a substantial, direct and intellectual contribution to the work, and approved it for publication.

### Conflict of Interest Statement

The authors declare that the research was conducted in the absence of any commercial or financial relationships that could be construed as a potential conflict of interest.
